# MyProteinNet: build up-to-date protein interaction networks for organisms, tissues and user-defined contexts

**DOI:** 10.1093/nar/gkv515

**Published:** 2015-05-18

**Authors:** Omer Basha, Dvir Flom, Ruth Barshir, Ilan Smoly, Shoval Tirman, Esti Yeger-Lotem

**Affiliations:** 1 Department of Clinical Biochemistry & Pharmacology, Faculty of Health Sciences, Ben-Gurion University of the Negev, Beer-Sheva 84105, Israel; 2 Department of Computer Science, Faculty of Natural Sciences, Ben-Gurion University of the Negev, Beer-Sheva 84105, Israel; 3 National Institute for Biotechnology in the Negev, Ben-Gurion University of the Negev, Beer-Sheva 84105, Israel

## Abstract

The identification of the molecular pathways active in specific contexts, such as disease states or drug responses, often requires an extensive view of the potential interactions between a subset of proteins. This view is not easily obtained: it requires the integration of context-specific protein list or expression data with up-to-date data of protein interactions that are typically spread across multiple databases. The MyProteinNet web server allows users to easily create such context-sensitive protein interaction networks. Users can automatically gather and consolidate data from up to 11 different databases to create a generic protein interaction network (interactome). They can score the interactions based on reliability and filter them by user-defined contexts including molecular expression and protein annotation. The output of MyProteinNet includes the generic and filtered interactome files, together with a summary of their network attributes. MyProteinNet is particularly geared toward building human tissue interactomes, by maintaining tissue expression profiles from multiple resources. The ability of MyProteinNet to facilitate the construction of up-to-date, context-specific interactomes and its applicability to 11 different organisms and to tens of human tissues, make it a powerful tool in meaningful analysis of protein networks. MyProteinNet is available at http://netbio.bgu.ac.il/myproteinnet.

## INTRODUCTION

Physical interactions between proteins underlie cellular pathways in all living organisms. The physical interactions between protein kinases and their substrates in signal transduction pathways (1), and the physical interactions between chaperones and their client proteins in protein homeostasis processes (2), are just two examples. Therefore, an important step in elucidating cellular pathways has been the mapping of all possible physical interactions between proteins. Mapping efforts have been conducted in model organisms (3–5) and in human (6–8), and resulted in several thousands of protein–protein interactions (PPIs) per organism. Those PPIs are commonly denoted as the organism's interactome and represented as a network where nodes represent proteins and edges represent their PPIs (9,10). Analyses of such interactomes were successfully applied to illuminate the molecular basis of disease (8) and to identify disease genes (11).

Several challenges arise upon trying to meaningfully analyze interactomes. Firstly, in accordance with their recognized importance, experimentally detected PPIs are available for download in multiple databases. Yet no single database contains all known PPIs. Some databases contain manually-curated PPIs, e.g. SPIKE (12) and KEGG (13), while other databases contain PPIs identified via high-throughput experiments, such as BioGrid (14) and DIP (15). Secondly, different detection methods have varying reliabilities and biases, even upon focusing on experimentally-detected PPIs. The yeast two-hybrid method, for example, is suited for detecting potential binary interactions between proteins (16). Affinity-purification mass-spectrometry is suited for detecting co-complex PPIs and less so for capturing transient interactions (17). Lastly, the detection of PPIs in some generic condition may not represent what is happening within cells *in vivo*, even in cases where *in vitro* evidence is plentiful. The *in vivo* occurrence depends on additional factors, such as co-expression and co-localization of the interaction pair-mates.

Several approaches were developed in the field of interactome analysis to cope with these challenges. To enable an extensive view of an interactome, PPI data are often integrated and consolidated from multiple databases (18–20). To overcome the varying reliability of different detection methods, scoring schemes have been devised that associate interactions with scores that denote their reliability, often favoring PPIs that were detected by multiple methods (21–25). To better model specific cellular states, PPIs have been filtered based on co-expression (25–27) or co-localization of the interacting proteins (28). In support of these efforts, recent studies showed that such refined interactomes outperformed a generic interactome in predicting disease-associated genes (24,29).

Several web servers facilitate interactome analysis by offering the schemes specified above. Yet, each of the current web servers is limited in some important aspect (Table 1). The STRING database (18) and the APID web server (20) consolidate PPIs from multiple databases and assign scores to them, yet do not provide users with the ability to select PPI databases or to filter PPIs, except by protein names. The PSIQUIC View web server (19) allows users to select multiple databases from which PPI data will be gathered, yet does not consolidate PPIs or filters them. The HIPPIE database (30) allows users to download PPI data and filter them by tissue, but limits the download to a subset of up to 100 genes at a time. The IntScore web server (31) allows users to score PPIs according to seven different weighting schemes, however users provide their own list of PPIs. The TissueNet database (32) provides PPIs between human proteins that were filtered by tissue expression profiles, yet interactions are not scored and data are not automatically updated.

**Table 1.** List of related PPI resources

Resource	URL	Supported Features	Limitations
APID	http://bioinfow.dep.usal.es/apid/	Integrative database, PPIs are scored	Resources are not selectable; filtering limited to gene names
HIPPIE	http://cbdm.mdc-berlin.de/tools/hippie/	Integrative human PPI database, filters by tissue	PPI retrieval limited to 100 genes
IntScore	http://intscore.molgen.mpg.de/	Scores PPIs according to seven different weighting schemes	Limited to user-provided PPIs
PSICQUIC View	http://www.ebi.ac.uk/Tools/webservices/psicquic/view/main.xhtml	Resources are selectable	No consolidation or filtering of PPIs
STRING	http://string-db.org/	Integrative database, PPIs are scored	Resources are not selectable; filtering limited to gene names
TissueNet	http://netbio.bgu.ac.il/tissuenet/	Integrative human tissue PPI database	Fixed set of PPIs, no scoring

Here we present MyProteinNet, an integrative web server that allows users to construct refined interactomes for 11 different organisms. MyProteinNet integrates up-to-date PPI data from a user-selected subset of 11 PPI databases. It consolidates experimentally-detected PPIs from the different databases and scores interactions by their reliability, thus constructing a generic interactome. MyProteinNet allows users to filter this generic interactome by contexts, including gene ontology (GO) annotations (33) and molecular expression profiles. These expression profiles are provided by the user, or, in case of human tissues, are also provided by MyProteinNet. The output of MyProteinNet consists of commonly-used network measurements of the resulting generic and filtered interactomes, as well as downloadable files detailing their PPIs. The format of these output files matches that of other interactome analysis tools, such as Cytoscape (34) and ResponseNet (35), thus facilitating further interactome analysis. The ability of MyProteinNet to overcome major obstacles in interactome construction makes it a powerful tool for obtaining refined, context-specific interactomes.

## MATERIALS AND METHODS

### PPI data gathering

PPIs are downloaded from 11 supported PPI databases for 11 supported species every fortnight, using PSICQUIC interface (19). Protein identifiers are converted to Ensembl gene identifiers using Ensembl Biomart (36). In case proteins have more than one matching Ensembl gene identifier, corresponding PPIs are multiplied. The usage of PSICQUIC interface guarantees that every downloaded PPI record specifies the methods by which the interaction was detected, in PSI-MITAB format. To filter out PPIs that lack proper experimental validation, MyProteinNet scans each PPI record: if the record consists of a method considered as reliable experimental validation the PPI is gathered, otherwise the PPI is excluded from our dataset. The methods considered by MyProteinNet as reliable experimental validation and their PSI-MI identifiers are listed in Supplementary Table S1. Upon integrating data of PPIs from multiple resources following a user's request, the record of each PPI is unified to include all of its reliable validation methods in a non-redundant manner (i.e. a PSI-MI identifier is recorded at most once per interaction to ignore overlapping evidence). This information per PPI appears in the output interactome files.

### PPI scoring

**Figure 1.**
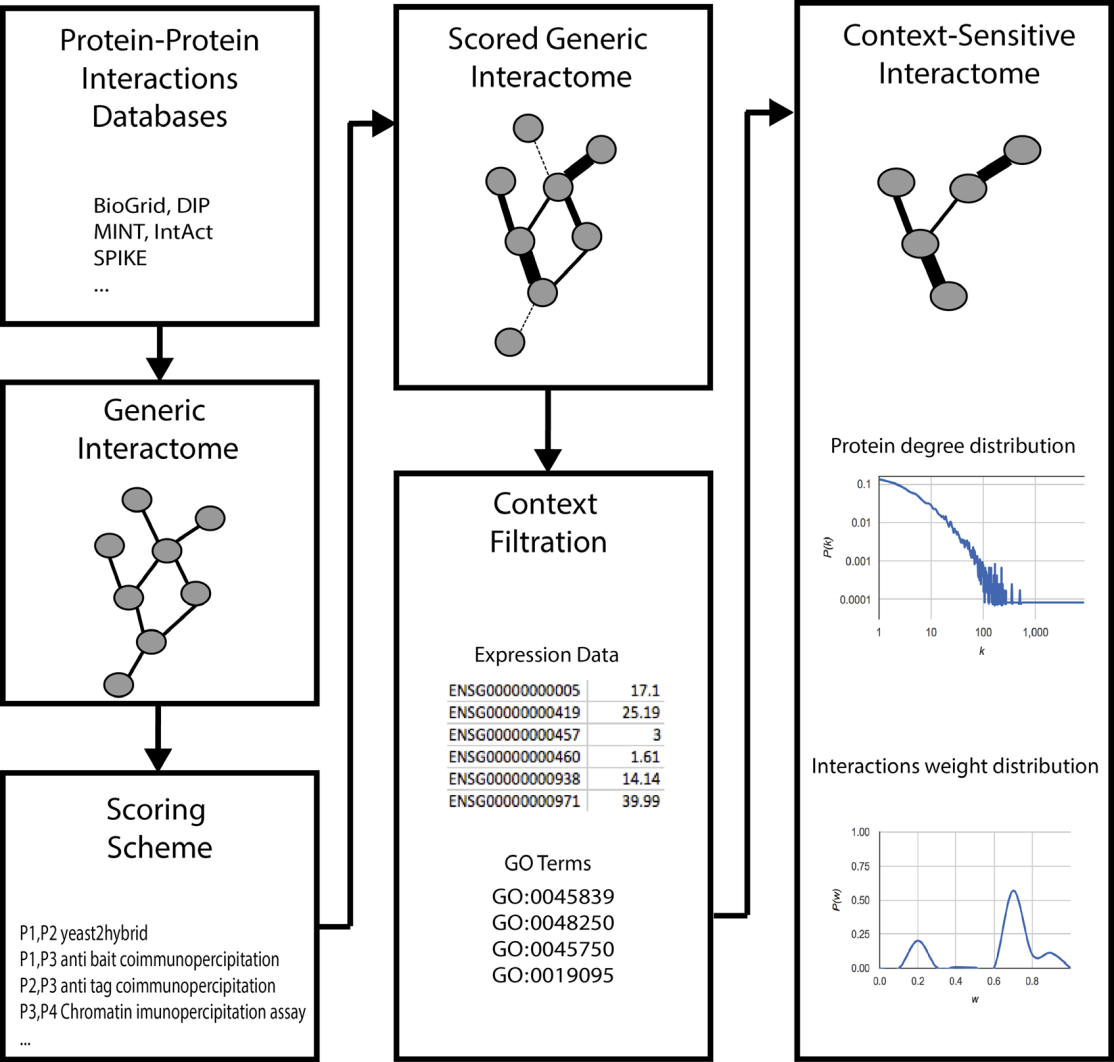
A flow chart of MyProteinNet analysis. The user selects databases, PPI scoring scheme and cellular context. Based on this selection, MyProteinNet constructs a generic interactome containing all mapped PPIs, scores each PPI based on the detection method(s) and then filters PPIs. This results in a weighted, context-sensitive interactome that is accompanied by its characteristic network measurements.

MyProteinNet uses a Bayesian scoring scheme published previously (22,35). This scheme works in two steps. In the first step, given a specific subset of GO terms (33), e.g. response pathways, the scheme creates positive and negative sets of PPIs as follows. The positive set includes PPIs between proteins annotated to these GO terms and the negative set includes PPIs between proteins that were not annotated to these GO terms. The score of each detection method is then computed based on the ratios of positive to negative PPIs that the detection method identified. In the second step the weight of each PPI is calculated according to the detection methods that identified it using a Bayesian computation (21). Since some detection methods identify only few PPIs, these methods and the corresponding PPIs cannot be scored reliably. To overcome this limitation, we manually combined detection methods that differ only slightly. For example, anti bait coimmunopercipitation (MI:0006) and anti tag coimmunopercipitation (MI:0007) were both grouped as coimmunopercipitation methods. MyProteinNet thus scores 37 combined detection methods (Supplementary Table S2). A diagram describing the distribution of PPI weights is provided as part of the output (Figure 1).

### GO terms download and selection

GO term annotations for every gene are downloaded automatically in every run using MyGene.info web service (37). To simplify GO term selection by the user, MyProteinNet offers 16 GO keywords. These keywords were carefully selected to reflect common GO terms: each keyword represented a word or part of word that appears in the GO term description (suffixes and prefixes were removed). We prioritized keywords by the number of different GO terms in which they appeared and listed the top 16 keywords in MyProteinNet input screen as check boxes to facilitate their selection. The user can also specify other GO terms, an option that is facilitated by an auto-complete search field.

### Expression data sources

MyProteinNet supports human tissue expression profiles from three resources measuring transcript levels (38) and Illumina Body Map 2.0 (39) or protein levels (HPA data version 8 (40). The processing of these data is described in detail elsewhere (41). While correlations between expression levels measured by different platforms were shown to be significant (32,42), the normalization of signals across platforms is an open issue and we do not aim to solve it here. Instead, MyProteinNet allows users to treat each resource individually by applying a threshold independently to each resource. Once a gene has a value above the defined threshold in the corresponding resource, this gene is considered as expressed and its interactions may be considered, depending on the expression of its interaction partners.

### Output file formats

The output consists of the generic interactome and the filtered interactome. PPIs in each interactome are scored and accompanied by the methods by which they were detected. PPIs are detailed in two separate files, one using Ensembl gene identifiers and the other using HUGO gene identifiers obtained via Ensembl Biomart (36).

## RESULTS

The MyProteinNet web server enables the user to construct up-to-date, weighted, context-specific interactomes for 11 different organisms. This is achieved in a series of steps in which current data of experimentally-detected PPIs within a specific organism are gathered, scored and filtered. A schematic flow diagram appears in Figure 1.

### Integrating current PPI data from multiple databases

MyProteinNet supports interactome construction for model organisms, such as *Saccharomyces cerevisiae* and *Drosophila melanogaster*, as well as for *Homo sapiens*. The user can select to gather PPI data from a subset of 11 major PPI databases (Table 2), all of which support the PSICQUIC interface for data download (19). Note that while the databases are heterogeneous and contain interactions with varying evidence types, MyProteinNet limits interactions only to physical interactions which have been validated experimentally. This filtering step is possible since the PSICQUIC interface guarantees that every database provides a record, per interaction, of the methods by which this interaction was detected. Thus, if the record of a certain PPI does not specify a method that is considered as experimental validation, this PPI is ignored. The list of methods considered by MyProteinNet as experimental validation appears in Supplementary Table S1. To allow MyProteinNet to consolidate data per user in a reasonable run time, PPI data are downloaded from all supported databases automatically every fortnight. Once the user selects a subset of databases, the relevant PPIs are integrated, such that each PPI is associated with the set of experimental methods by which it was detected.

**Table 2.** List of PPI databases supported by MyProteinNet

Database	URL	Organisms and features
BioGrid	http://thebiogrid.org	*Homo Sapiens, Caenorhabditis elegans, Drosophila melanogaster, Danio rerio, Gallus gallus, Sus scrufa, Bos taurus, Oryctolagus cuniculus, Mus musculus, Rattus norvegicus, Saccharomyces cerevisiae*; distinguishes high-throughput assays
DIP	http://dip.doe-mbi.ucla.edu/dip/	*H.Sapiens, C.elegans, D.melanogaster, S.scrufa, B.taurus, O.cuniculus, M.musculus, R.norvegicus, S.cerevisiae*; curated PPIs
InnateDB	http://www.innatedb.com/	*H.sapiens, M.musculs*; PPIs involved in innate immune response
IntAct	http://www.ebi.ac.uk/intact/	*H.Sapiens, C.elegans, D.melanogaster, G.gallus, S.scrufa, B.taurus, M.musculus, R.norvegicus, S.cerevisiae*; curated PPIs & direct user submissions
InteroPorc	http://biodev.extra.cea.fr/interoporc/	*H.sapiens, C.elegans, D.melanogaster, D.rerio, G.gallus, B.taurus, M.musculus, R.norvegicus, S.cerevisiae*; validated & predicted PPIs
MatrixDB	http://matrixdb.ibcp.fr/	*H.sapiens;* PPIs involving extracellular proteins
MINT	http://mint.bio.uniroma2.it/mint/	*H.Sapiens, C.elegans, D.melanogaster, D.rerio, G.gallus, B.taurus, O.cuniculus, M.musculus, R.norvegicus, S.cerevisiae*; curated PPIs
SPIKE	http://www.cs.tau.ac.il/∼spike/	*H.sapiens*; manually-curated pathway database
STRING	http://string-db.org	*H.Sapiens, C.elegans, D.melanogaster, D.rerio, G.gallus, S.scrufa, B.taurus, O.cuniculus, M.musculus, R.norvegicus, S.cerevisiae*; Integrative PPI database
TopFind	http://clipserve.clip.ubc.ca/topfind	*H.sapiens, M.musculus;* PPIs between proteases and substrates
Uniprot	http://www.uniprot.org	H.Sapiens, C.elegans, D.melanogaster, G.gallus, B.taurus, M.musculus, R.norvegicus, S.cerevisiae; Protein-centric resource, high-confidence PPIs selected from IntAct

### Scoring the interactome

MyProteinNet scores PPIs by using an established Bayesian scoring scheme that takes into account the type of methods by which interactions were detected, as well as the GO annotations of the interacting proteins (22). In the first step, detection methods are scored by their ability to detect PPIs involving proteins annotated to specific GO terms. In the second step, each PPI is scored according to the scores of its detection methods (see ‘Materials and Methods’ section). MyProteinNet users can select the GO terms to be used in the first step. This way, users can bias scores to favor, e.g. PPIs between proteins involved in signaling or between proteins localized to the mitochondria. The user can limit GO terms by size, so as to ignore terms that are too broad. The user can also limit proteins by the evidence codes of their GO annotation, to increase the confidence of the GO assignment and thus enhance the reliability of the scoring scheme.

### Filtering the interactome by context

The interactome created in previous steps is oblivious to the actual cellular state or process to be modeled and thus may contain PPIs that are irrelevant in this context. MyProteinNet enables users to construct a context-sensitive interactome by filtering the generic interactome. Since a PPI cannot occur if any of the two interacting proteins is missing, the first filtering option is by the expression of the two interacting proteins in the studied condition. To apply this filter, the user can upload an expression profile in the form of a table specifying gene name, and/or expression level and/or *P*-value, and specify threshold values. MyProteinNet will filter the interactome, such that a PPI will be included only if both pair-mates obey the user-defined thresholds. In addition, MyProteinNet provides extensive support for human tissue interactome analysis: it maintains tissue expression profiles from three major data sources pertaining to 16 main human tissues and around 80 sub-parts (38,43,44). The user can select any subset of these data sources, define an independent threshold for each data source, and thus easily obtain a tissue-sensitive interactome. An additional filtering option is by GO annotations. Users can enter a list of selected GO terms and only interactions where both pair-mates are associated with GO terms in the input list, will be included in the output interactome. For example, by filtering for mitochondria-related cellular component GO terms, the user can focus on PPIs occurring in the mitochondria and by filtering for chaperones the user can focus on protein-homeostasis processes.

### MyProteinNet output

The output of MyProteinNet consists of downloadable interactome files, detailing the interactions composing the generic interactome and the filtered context-specific interactome (Figure 1). The web server also presents a summary table listing various measurements of the output interactomes, such as the number of proteins, PPIs, connected components and average clustering coefficient. Plots describing the node degree distribution and the PPI score distribution are also provided. A typical run takes ∼9 min for the relatively rich yeast and human interactomes and is generally faster for other organisms for which interaction data are sparser. A log of user sessions is maintained by the server for three months to support users wishing to retrieve their analysis.

## DISCUSSION

The analysis of interactomes, especially in the context of human disease, is becoming more and more common (45,46). The MyProteinNet web server facilitates much of the effort needed in order to construct up-to-date interactomes that are also context-sensitive. Users can upload expression data and GO terms, and create interactomes that are not only current and extensive, but also meaningful for the analysis of specific cellular states or processes. An important focus of MyProteinNet is the analysis of human tissue interactomes. Our knowledge of PPIs among human proteins is mounting (8) and our view of the molecular expression in human tissues is increasing fast across tissues, proteins and individuals (47–50). Moreover, tissue interactome analyses were shown recently to have particular value, beyond that of generic interactomes, in predicting disease genes (23,24) and disease mechanisms (41). Thus, we anticipate that tools like MyProteinNet that facilitate interactome analysis and open it to researchers that are not hard-core bioinformaticians, will greatly contribute to the understanding of cellular, tissue and disease processes.

## AVAILABILITY

MyProteinNet: http://netbio.bgu.ac.il/myproteinnet.

## SUPPLEMENTARY DATA

Supplementary Data are available at NAR Online.

SUPPLEMENTARY DATA
