# Supplementary material for: MyProteinNet: build up-to-date protein interaction networks for organisms, tissues and user-defined contexts
Source: Nucleic Acids Res. 2015 May 18;43(Web Server issue):W258–63. doi: 10.1093/nar/gkv515 (PMC4489290; doi:10.1093/nar/gkv515)
Supplement: SUPPLEMENTARY DATA [file supp_43_W1_W258__index.html]

MyProteinNet: build up-to-date protein interaction networks for organisms, tissues and user-defined contexts — MyProteinNet: build up-to-date protein interaction networks for organisms, tissues and user-defined contexts — SUPPLEMENTARY DATA 

# MyProteinNet: build up-to-date protein interaction networks for organisms, tissues and user-defined contexts

## SUPPLEMENTARY DATA

- SUPPLEMENTARY DATA
