# Supplementary material for: MyProteinNet: build up-to-date protein interaction networks for organisms, tissues and user-defined contexts
Source: Nucleic Acids Res. 2015 May 18;43(Web Server issue):W258–63. doi: 10.1093/nar/gkv515 (PMC4489290; doi:10.1093/nar/gkv515)
Supplement: SUPPLEMENTARY DATA [file supp_gkv515_nar-00634-web-b-2015-File003.pdf]

Supplementary Information

MyProteinNet: Build up-to-date protein interaction networks for organisms, tissues and user-defined contexts

Omer Basha[1], Dvir Flom[2], Ruth Barshir[1], Ilan Smoly[2], Shoval Tirman[1] & Esti Yeger-Lotem[1,3,*]

[1]Department of Clinical Biochemistry & Pharmacology, Faculty of Health Sciences; [2]Department of Computer Science, Faculty of Natural Sciences; and [3]National Institute for Biotechnology in the Negev, Ben-Gurion University of the Negev, Beer-Sheva 84105, Israel

* Corresponding author: estiyl@bgu.ac.il

MyProteinNet: http://netbio.bgu.ac.il/myproteinnet

This file contains the following Tables:

Supplementary Table 1. Detection methods considered by MyProteinNet as experimental validation for physical interaction.

Supplementary Table 2. The grouping of detection methods by type (for MyProteinNet's PPI scoring scheme).

Supplementary Table 1: Detection methods considered by MyProteinNet as experimental validation for physical interaction.

PSI-MITAB Identifier	Method Name
MI:0004	affinity chromatography technology
MI:0006	anti bait coimmunoprecipitation
MI:0007	anti tag coimmunoprecipitation
MI:0009	bacterial display
MI:0010	beta galactosidase complementation
MI:0011	beta lactamase complementation
MI:0012	bioluminescence resonance energy transfer
MI:0013	biophysical
MI:0014	adenylate cyclase complementation
MI:0016	circular dichroism
MI:0017	classical fluorescence spectroscopy
MI:0018	two hybrid
MI:0019	coimmunoprecipitation
MI:0020	transmission electron microscopy
MI:0030	cross-linking study
MI:0031	protein cross-linking with a bifunctional reagent
MI:0038	dynamic light scattering
MI:0040	electron microscopy
MI:0041	electron nuclear double resonance
MI:0042	electron paramagnetic resonance
MI:0043	electron resonance
MI:0047	far western blotting
MI:0049	filter binding
MI:0051	fluorescence technology
MI:0052	fluorescence correlation spectroscopy
MI:0053	fluorescence polarization spectroscopy
MI:0054	fluorescence-activated cell sorting
MI:0055	fluorescent resonance energy transfer
MI:0065	isothermal titration calorimetry
MI:0067	light scattering
MI:0069	mass spectrometry studies of complexes
MI:0071	molecular sieving
MI:0077	nuclear magnetic resonance
MI:0090	protein complementation assay
MI:0091	chromatography technology
MI:0096	pull down
MI:0097	reverse ras recruitment system

MI:0099	scintillation proximity assay
MI:0104	static light scattering
MI:0107	surface plasmon resonance
MI:0111	dihydrofolate reductase reconstruction
MI:0112	ubiquitin reconstruction
MI:0114	x-ray crystallography
MI:0115	yeast display
MI:0225	chromatin immunoprecipitation array
MI:0226	ion exchange chromatography
MI:0227	reverse phase chromatography
MI:0231	mammalian protein protein interaction trap
MI:0232	transcriptional complementation assay
MI:0276	blue native page
MI:0369	lex-a dimerization assay
MI:0370	tox-r dimerization assay
MI:0397	two hybrid array
MI:0398	two hybrid pooling approach
MI:0399	two hybrid fragment pooling approach
MI:0402	chromatin immunoprecipitation assay
MI:0404	comigration in non denaturing gel electrophoresis
MI:0410	electron tomography
MI:0411	enzyme linked immunosorbent assay
MI:0412	electrophoretic mobility supershift assay
MI:0413	electrophoretic mobility shift assay
MI:0416	fluorescence microscopy
MI:0417	footprinting
MI:0419	gtpase assay
MI:0420	kinase homogeneous time resolved fluorescence
MI:0423	in-gel kinase assay
MI:0424	protein kinase assay
MI:0425	kinase scintillation proximity assay
MI:0426	light microscopy
MI:0428	imaging technique
MI:0430	nucleic acid uv cross-linking assay
MI:0432	one hybrid
MI:0434	phosphatase assay
MI:0437	protein three hybrid
MI:0509	phosphatase homogeneous time resolved fluorescence
MI:0510	homogeneous time resolved fluorescence
MI:0511	protease homogeneous time resolved fluorescence
MI:0514	in gel phosphatase assay

MI:0515	methyltransferase assay
MI:0516	methyltransferase radiometric assay
MI:0588	three hybrid
MI:0602	chemical footprinting
MI:0603	dimethylsulphate footprinting
MI:0604	potassium permanganate footprinting
MI:0605	enzymatic footprinting
MI:0606	DNase I footprinting
MI:0655	lambda repressor two hybrid
MI:0663	confocal microscopy
MI:0676	tandem affinity purification
MI:0726	reverse two hybrid
MI:0727	lexa b52 complementation
MI:0728	gal4 vp16 complementation
MI:0729	luminescence based mammalian interactome mapping
MI:0807	comigration in gel electrophoresis
MI:0808	comigration in sds page
MI:0809	bimolecular fluorescence complementation
MI:0809	bimolecular fluorescence complementation
MI:0814	protease accessibility laddering
MI:0824	x-ray powder diffraction
MI:0825	x-ray fiber diffraction
MI:0826	x ray scattering
MI:0827	x-ray tomography
MI:0841	phosphotransferase assay
MI:0858	immunodepleted coimmunoprecipitation
MI:0859	intermolecular force
MI:0872	atomic force microscopy
MI:0879	nucleoside triphosphatase assay
MI:0880	atpase assay
MI:0888	small angle neutron scattering
MI:0891	neutron fiber diffraction
MI:0892	solid phase assay
MI:0893	neutron diffraction
MI:0894	electron diffraction
MI:0895	protein kinase A complementation
MI:0901	isotope label footprinting
MI:0905	amplified luminescent proximity homogeneous assay
MI:0916	lexa vp16 complementation
MI:0921	surface plasmon resonance array
MI:0928	filter trap assay

MI:0938	rheology measurement
MI:0943	detection by mass spectrometry
MI:0944	mass spectrometry study of hydrogen/deuterium exchange
MI:0947	bead aggregation assay
MI:0963	interactome parallel affinity capture
MI:0964	infrared spectroscopy
MI:0965	2d-infrared spectrometry
MI:0966	ultraviolet-visible spectroscopy
MI:0968	biosensor
MI:0969	bio-layer interferometry
MI:0976	total internal reflection fluorescence spectroscopy
MI:0979	oxidoreductase assay
MI:0982	electrophoretic mobility-based method
MI:0983	gemma
MI:0984	deaminase assay
MI:0989	amidase assay
MI:1000	hydroxylase assay
MI:1001	lipidase assay
MI:1002	myristoylase assay
MI:1003	geranylgeranylase assay
MI:1004	palmitoylase assay
MI:1005	adp ribosylase assay
MI:1006	deglycosylase assay
MI:1008	sumoylase assay
MI:1009	desumoylase assay
MI:1016	fluorescence recovery after photobleaching
MI:1017	rna immunoprecipitation
MI:1019	protein phosphatase assay
MI:1024	scanning electron microscopy
MI:1026	diphtamidase assay
MI:1028	modified chromatin immunoprecipitation
MI:1029	proteomics of isolated chromatin segments
MI:1030	excimer fluorescence
MI:1037	Split renilla luciferase complementation
MI:1038	silicon nanowire field-effect transistor
MI:1086	equilibrium dialysis
MI:1103	solution state nmr
MI:1104	solid state nmr
MI:1111	two hybrid bait or prey pooling approach
MI:1112	two hybrid prey pooling approach
MI:1113	two hybrid bait and prey pooling approach

MI:1137	carboxylation assay
MI:1138	decarboxylation assay
MI:1142	aminoacylation assay
MI:1145	phospholipase assay
MI:1147	ampylation assay
MI:1183	nuclease footprinting
MI:1189	methylation interference assay
MI:1190	hydroxy radical footprinting
MI:1191	ultraviolet (uv) footprinting
MI:1203	split luciferase complementation
MI:1204	split firefly luciferase complementation
MI:1218	chromosome conformation capture assay
MI:1219	enzyme-mediated activation of radical sources
MI:1229	uridylation assay
MI:1235	thermal shift binding
MI:1236	proline isomerase assay
MI:1238	mass spectrometry studies of subunit exchange
MI:1246	ion mobility mass spectrometry of complexes
MI:1249	isomerase assay
MI:1252	methylmalonyl-CoA isomerase asf say

Supplementary Table 2. The grouping of detection methods by type (for MyProteinNet's PPI scoring scheme).

Assay type	PSI-MITAB Identifiers
2-hybrid	MI:0018,MI:0397,MI:0727,MI:0728,MI:0398,MI:1113,MI:1111, MI:1112,MI:0399
ADP-ribosylase	MI:1005
Amidase	MI:0989
Aminoacylation	MI:1142
Ampylation	MI:1147
Biophysical	MI:0013,MI:0859,MI:0938,MI:0065,MI:0067,MI:0104,MI:0038, MI:0826,MI:0891,MI:0893,MI:0016,MI:1235,MI:1311,MI:1219, MI:0894,MI:0905,MI:0964,MI:0965,MI:0966,MI:0968,MI:0107, MI:0921,MI:1038,MI:0969,MI:0043,MI:0042,MI:0041,MI:1247, MI:0099,MI:0888,MI:1086
Carboxylation	MI:1137
Chromatography	MI:0091,MI:0227,MI:0004,MI:0096,MI:0963,MI:0676,MI:0928, MI:0226,MI:0071
Co-immunopercipitation	MI:0019,MI:0402,MI:1218,MI:1028,MI:0225,MI:1029,MI:0858, MI:0946,MI:0007,MI:0006,MI:1017
Complementation	MI:0090,MI:0895,MI:0097,MI:0370,MI:0014,MI:0111,MI:0112, MI:0010,MI:0011,MI:0809,MI:0232,MI:0588,MI:0369,MI:0726, MI:0655,MI:0432,MI:0437,MI:0916,MI:1203,MI:1204,MI:1037
Crosslink	MI:0030,MI:0430,MI:0031
de-ADP	MI:1309
Deaminase	MI:0984
Decarboxylation	MI:1138
Deglycosylase	MI:1006
Desumoylase	MI:1009
Diphtamidase	MI:1026
Electrophoresis	MI:0982,MI:0807,MI:0404,MI:0276,MI:0413,MI:0412,MI:1312, MI:0808,MI:0983
Fluorescence	MI:0051,MI:0012,MI:1030,MI:0055,MI:0017,MI:0809,MI:1016, MI:0052,MI:0053,MI:0054,MI:0115,MI:0009,MI:0510,MI:0509, MI:0511,MI:0420,MI:0976
Footprinting	MI:0417,MI:1191,MI:0605,MI:0814,MI:1189,MI:1183,MI:0606, MI:0901,MI:0602,MI:1190,MI:0603,MI:0604
Hydroxylase	MI:1000
Isomerase	MI:1249,MI:1236,MI:1252
Lipidase	MI:1001,MI:1004,MI:1002,MI:1003
Lumier	MI:0729
Mappit	MI:0231
Methyltransferase	MI:0515,MI:0516
Microscopy	MI:0428,MI:0426,MI:0872,MI:0663,MI:0416,MI:0040,MI:0410, MI:0020,MI:1024
MS-interact	MI:0943,MI:0069,MI:1246,MI:1238,MI:0944
NMR	MI:0077,MI:1104,MI:1103

Oxidoreductase	MI:0979
Phosphatase	MI:0434,MI:0514,MI:0879,MI:0419,MI:0880,MI:1019
Phospholipase	MI:1145
Phosphotransferase	MI:0841,MI:0424,MI:0425,MI:0423
solid-phase	MI:0892,MI:0411,MI:0049,MI:0047,MI:0947
Sumoylase	MI:1008
Uridylation	MI:1229
X-ray	MI:0114,MI:0825,MI:0824,MI:0827
